# Osteo-Sarcoma below Trochanter Major: Resection in the Continuity; Cure

**Published:** 1887-02

**Authors:** B. E. Hadra

**Affiliations:** Austin, Texas


### OSTEO-SARCOMA BELOW TROCHANTER MAJOR. RESECTION IN THE CONTINUITY; CURE.

*By B. E. Hadra, M. D., Austin, Texas.*

For Daniel's Texas Medical Journal.

Harry Cohn, 19 years old, German Israelite, had suffered a fracture of the left thigh, right under the trochanter major about 7 years ago. The fracture healed with about 1½ inch shortening and a deviation of the lower end upwards and posteriorly to the upper end, as the surgeons who attended the case, state. It was therefore a somewhat rectangular projection formed, the upper end taking a nearly horizontal position. The patient got along with this, though not splendid, still under the difficult circumstances (short upper part) satisfactory result, till some five years after the accident, the projections on the hip seemed to enlarge ; but it was not before the next year that direct discomfort was experienced. Then, about October, 1885, there existed constant dull pain, and a great weakness of the extremity, to such an extent that he could not stand on it, or if he was sitting, could not rise without assistance.

The patient consulted me in January 1886, when there was a large (½) half-globe formed, but dense and firm projection of about the size of two fists to be found, bulging out most laterally under the large trochanter, which seemed to form the upper part of the tumor. I must now candidly conceed that the hardness of the tumor made me believe, that it was an extrordinarly smooth and rounded Exostosis, or rather an ossified callous mass, due to the fracture, especially as there were no enlarged inguinal glands. The patient was advised to undergo an operation, to which he consented.

On the 17th of January 1886, then with the assistance of Drs. DuPre, J. W. and F. P. McLaughlin and W. J. Mathews I proceeded to perform a wedge shaped exsection, with the view to break in the thigh and to unite the ends. The incision through the skin over the height of the protrusion exposed at once a white glistening bony surface. There was hardly any connective tissue between the bone and its covers The chisel fell at once, after having penetrated a bony shell of about ⅓ of an inch thickness, into a semi-soft mass and, of course, it was evident that the diagnosis had not been correct. The whitish looking mass in appearance and consistency very much like the pulp of cocoanut, formed a bulbous mass of about 3 inches diameter, and branched out in 2 or 3 processes into the shaft of the bone, I suppose into the lines of fracture. It was on both ends freely chiseled away enough to be sure of having removed all diseased bone and more. Wire seeming not strong enough to unite firmly the cut ends of the femur, a strong steel nail was driven in from above through the bones, after a canal had been drilled. It was only with difficulty that the upper horizontal end was held down to the other in proper direction, but after the nail once was driven in, no further inconvenience was experienced. The wound was then closed with catgut, only the nails' head allowed to pass, and a borated cotton padding applied. It is hardly necessary to state that all was done aseptically, though no spray was used. A plaster of paris bandage, taking in the pelvis and the whole extremity finished the operation, which lasted alltogether over two hours.

The patient did not do well in the beginning, he being of extreme nervosity, or better expressed a hysterical subject, was a constant complainer and lost so much the grip of self-control, the fœces and urine were passed under him. The gypsum bandage therefore had to be renewed nearly every 12th day. The wound did not do well either; excessive fungous granulations surrounded the nail and constant applications of nitrate of silver, iodoform etc. was required to keep them down until the 6th week the nail was removed, the bones having united, and the irritation of the foreign body being considered the main cause of these morbid granulations.

The cutaneous covers still forming a kind of a pocket, constant washings and drainage was required for 3 months, in which time all seemed to do well, and when it was considered safe to remove the gypsum bandage also.

Still for further 3 months the point formerly around the nail kept open, and in the seventh month, when a pocket seemed to have formed, an opening was established on the most depressing part of it, and a drainage tube through both openings carried through, after the inside had been well rubbed out with iodoform, the bones were then found healthy by touch. In October this sinus closed entirely and the patient is now, nearly a year after the operation, in perfect health, having his left leg shorter 4 inches, walking on an elevated sole and with the assistance of a cane. An angular protrusion of the bone is still present, evidently owing to an angular union, but not to any enlargement of the bone. He only occassionally suffers from rheumatoid pains when the weather changes, like it is usual in cases of fracture.

The most annoying and persistent complication was a bullous eruption, mostly on his lower left leg, where the remains in form of ulcers are yet present. There was altogether hardly a case so trying to the patience of the attendants, doctors and relatives as this, and I deem it my duty to express my thanks to Drs. McLaughlin and Mathews for their kind assistance.

The tumor was examined by Dr. J. W. McLaughlin, and this is what he found:

The tumor was principally composed of large round cells with an occasional giant cell imbedded in a fibro-cartilaginous stroma. Interspersed here and there throughout the tumor were to be found bony trabeculæ.

Reviewing the interesting case, I may be blamed for the incorrectness of the diagnosis, for which I do not apologize. At the same time this mistake saved the patient his leg, because otherwise I would not have hesitated to exarticulate the leg. I naturally refrained from doing so, after the mistake was discovered, because he, under anesthesia, conld not have given his consent to this extension of the operation. As the patient is free now nearly for a whole year from a recurrence of the disease, and as no enlarged glands can be detected in the neighborhood, and as his general health is perfectly good, a permanent cure may be considered probable. In regard to the tumor, its origin from the lines of fracture can hardly be doubted, The ætiology of it as well as the hard osseous shell prove that it was not of periosteal character. It may be considered of myologenous origin, but its processes into the fissures of the fracture make it doubtful also, though the tumor may have taken its start in the cavity and then grown between the fractured bones, or also the cavity may be considered so broken up and displaced as to having formed parts of the fracture lines.

